# Predictive model for persistent hypertension after surgical intervention of primary aldosteronism

**DOI:** 10.1038/s41598-023-39028-2

**Published:** 2023-07-22

**Authors:** Zhuoying Li, Yunfeng He, Yao Zhang, Gang Chen, Yongbo Zheng, Yuan Guo, Zhen Quan, Xiaohou Wu

**Affiliations:** 1 Department of Urology, The Ninth People’s Hospital of Chongqing, 69 Jialing Village, Beibei District, Chongqing, 400700 China; 2 Department of Urology, The First Affiliated Hospital of Chongqing Medical University, Yi Xue Yuan Road, Chongqing, 400016 China

**Keywords:** Endocrine system and metabolic diseases, Endocrinology, Oncology

## Abstract

Primary aldosteronism (PA) is one of the most common causes of secondary hypertension and is potentially curable. However, a large number of patients still undergo persistent hypertension (PHT) after unilateral adrenal surgery. This research retrospectively studied the factors associated with this clinical difficulty and established a prediction model for the postoperative PHT; Methods: 353 patients from 2014 to 2021 with PA undergoing unilateral adrenal surgery were enrolled in this study. Clinical and biochemical characteristics were reviewed and the associating factors were examined using univariate and multivariate analysis. A nomogram-based prediction model was established correspondingly; results: 46.2% (163/190) of patients had post-surgical PHT. Multivariate analysis suggested that BMI ≥ 25, diabetes, duration of hypertension, male gender, and ARR were independent predictors of PHT after surgery. The prediction model based on the nomogram showed good discrimination ability (the C index of the training group and the validation group were 0.783 and 0.769, respectively), and the calibration curves and the Hosmer–Lemeshow test were good as well. Clinical usefulness was quantified using the decision curve analysis; This nomogram is an integration of the clinical and biochemical data of patients before surgery, and is a reliable tool with high accuracy for predicting the postoperative PHT in patients with PA.

## Introduction

Primary aldosteronism (PA) is an abnormally high secretion of aldosterone of the adrenal cortex, leading to the increase of plasma aldosterone concentration, the inhibition of renin activity, and hence a syndrome, namely Conn syndrome, manifested with hypertension, hypokalemia, and metabolic alkalosis. PA is a common cause of secondary hypertension, often resulting in resistant hypertension (RH). Many previous reports have reported that when blood pressure is matched, patients with PA are more likely to suffer cardiovascular and cerebrovascular events and target organ damages due to the abnormal increased plasma aldosterone. In China, the prevalence of PA in hypertensive population is at least 4%^1^.

The main types of PA include: (1) idiopathic hyperaldosteronism (bilateral adrenal hyperplasia); (2) aldosterone-producing adenomas, APA; (3) unilateral adrenal hyperplasia, UNAH; (4) familial hyperaldosteronism, FH; (5) pure aldosterone-producing adrenocortical carcinoma, ACC^2^. Surgical removal of the tumor or the whole gland is generally employed in patients with APA or UNAH in hope that the associating clinical symptoms relieves along with the decrease of abnormally elevated aldosterone in plasma. Previous studies indicate that the most of patients with PA can achieve biochemical cure (83–100%) after surgery^3–5^. Among the 705 patients in the PASO study, 84% of them achieved either complete (37%) or partial (47%) clinical success after adrenalectomy^3^. In addition, previous publications reported that the complete cure rate was generally low (20–66%)^6–10^. Therefore, there are still a significant number of patients who remain dependent on antihypertensive medications after surgery, and it is important to identify this subgroup of patients. It could help the clinicians to better assess the postoperative clinical results and to strengthen the follow-up of the target population.

## Materials and methods

### Patient collection

**Figure 1:**
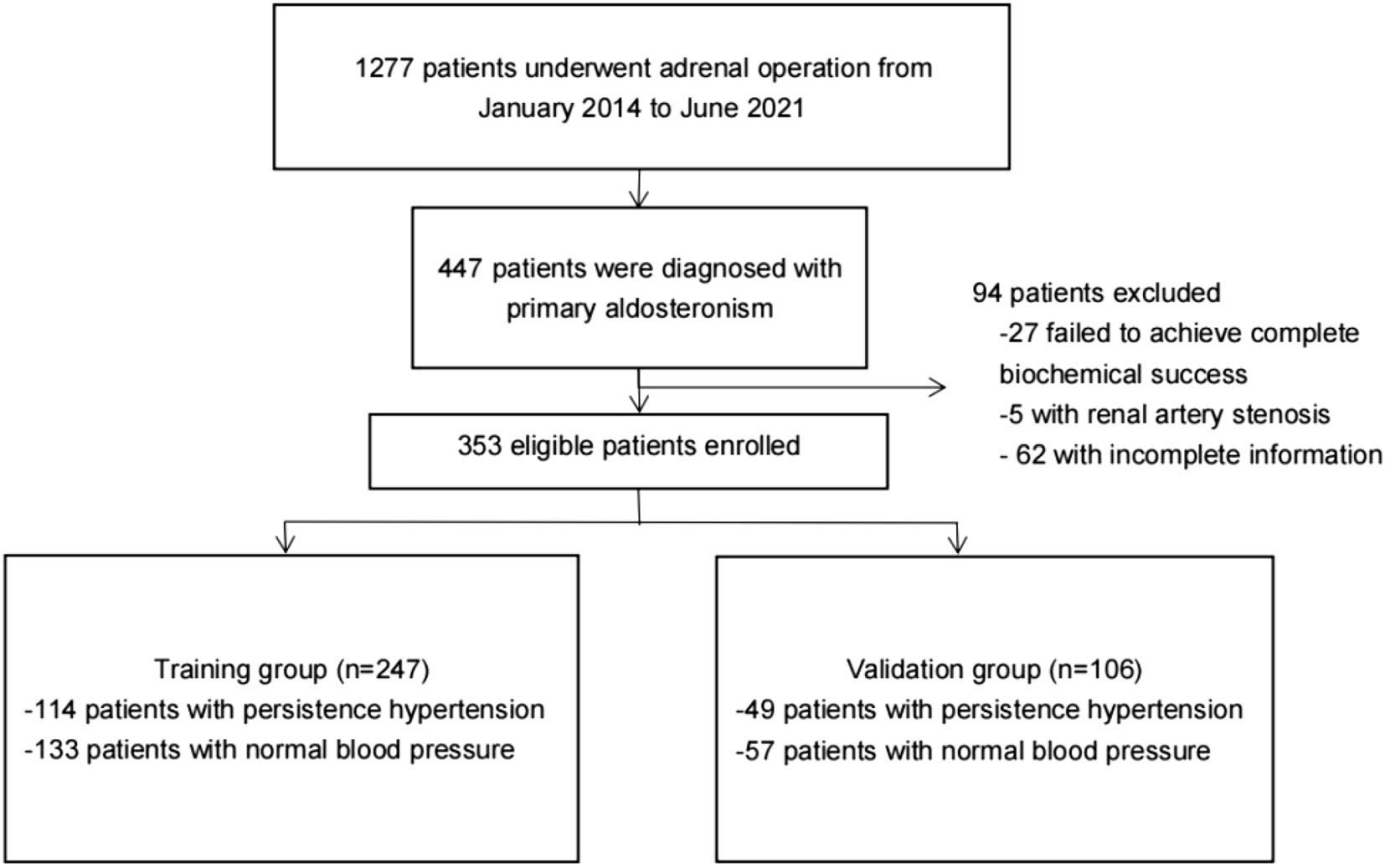
Flowchart.

This retrospective study was approved by local institutional ethics committee. Because there was no patient interest or privacy, informed consent was waived after careful review by Ethics Committee of the First Affiliated Hospital of Chongqing Medical University. A total of 1277 underwent adrenal operation at the Department of Urology, The First Affiliated Hospital of Chongqing Medical University, between January 2014 to June 2021. The criterion for inclusion were: (1) preoperative diagnosis of PA according to Endocrine Society guidelines; (2) preoperative diagnosis of hypertension (with or without hypokalemia); (3) perform unilateral adrenal surgery. Patient were excluded with any of following conditions: (1) those failed to achieve complete biochemical success after surgery; (2) suffering from other known diseases that can cause secondary hypertension; (3) those with incomplete clinical data or with a follow-up shorter than 6 months after discharge. A total of 353 eligible patients were ultimately included in this study (Fig. 1).

### Diagnosis and subtyping of PA

The diagnosis of PA is based on the Endocrine Society guidelines. Antihypertensive drugs interfering with detection should be withdrawn for at least 2 weeks before aldosterone/renin ratio (ARR) detection testing. Diuretics should be stopped at least 4 weeks (including mineralocorticoid receptor antagonist). The cut-off for detection test is a ARR higher than 20 pg mL^−1^/uIU mL^−1^. Patients with suspected PA must use at least one confirmatory test (captopril challenge test, saline infusion test, fludrocortisone suppression test)^2^. Patients with confirmed PA underwent computed tomography (CT) and adrenal venous samples (AVS) to locate the corresponding lesions. When patients presented with bilateral normal adrenal glands or bilateral adrenal lesions on CT, the final surgical side was determined based on the lateralization in AVS. Besides, part of patients with typical adenomas diagnosed by CT underwent surgical treatment directly without undergoing AVS. In our study, AVS was performed in 235 patients, while CT was conducted in another 118 patients. Among the patients diagnosed by CT, 43 patients were young (below 35 years old), and an additional 75 patients were pathological diagnosed typical adenomas. We excluded patients who did not achieve biochemical success to minimize the potential errors associated with using CT alone for diagnosis. Thus all the patients were diagnosed with unilateral PA. (Supplementary materials).

### Definition of HHD, diabetes, RH and eGFR

The hypertensive heart disease (HHD) is diagnosed with the history of hypertension and the heart dysfunction showed in cardiac color Doppler ultrasound examination. According to the 2015 American Diabetes Association, diabetes mellitus is defined as fasting blood glucose greater than 7.0 mmol/L or blood glucose greater than 11.1 mmol/L two hours after the OGTT experiment^11^. Resistant hypertension (RH) is defined as the inability to reduce blood pressure (BP) to the normal range with at least three or more antihypertensive drugs (including diuretics)^12^. Estimated glomerular filtration rate (eGFR) was used to evaluate the renal function, which was calculated by the Modification of Diet in Renal Disease (MDRD) formula combined with age, gender, and serum creatinine^13^. Due to the incomplete information of markers such as microalbuminuria and cystatin C in partial patients, these variables were not utilized in our study.

### Surgical procedure

Spironolactone (200–400 mg/d), irbesartan, nifedipine and other routine antihypertensive agents were used to lower BP during preoperative period (under 140/90 mmHg or 150/90 mmHg in routine patients or RH patients respectively). Oral or intravenous potassium supplementation was used to correct hypokalemia (if present), and to maintain serum potassium greater than 4.0 mmol/L before surgery. All patients underwent retroperitoneal laparoscopic surgery. ALL surgeries were performed by surgeons of equal seniority. Adrenal tumors or nodules were removed in surgery. However, when there was no obvious tumor or multiple tumors existed in adrenal gland, a total adrenalectomy was performed.

### Definition of postoperative outcome

We reviewed the complete medical records of all patients with PA undergoing adrenal surgery at the First Affiliated Hospital of Chongqing Medical University. According to PASO criteria, BP is assessed at 6 to 12 months after surgery. Patients were categorized as PHT if they had partial or absent clinical success, which means they still underwent hypertension or continued need antihypertensive medications to control BP. Normal BP postoperatively (complete clinical success) is defined as BP in the normal range (SBP < 140 mmHg and DBP < 90 mmHg) without taking any antihypertensive drugs. The detail methods of blood pressure measurement are described in supplementary materials.

### Statistical analysis

Date distributions were analyzed by Kolmogorov–Smirnov test. Normally distributed variables were expressed as mean ± SD and were analyzed by the student t test. Otherwise, it was expressed as median (quartile range), and Mann–Whitney U test was used for it. Logarithmic transformation was used for skewed distribution data to make it normal. Chi-square test or Fisher’s exact test was used to compare categorical variables. On the basis of univariate logistic regression, multicollinearity diagnostic method was carried out to eliminate variables with common causes and obvious intermediary variables. In R software, the sample.split function in the caTools of R package was used to divide the data set. All patients were divided into the training set (70%) or the validation set (30%). A nomogram predicting post-surgical PHT was established based on the results of univariate and multivariate analyses. Receiver operating characteristic (ROC) curve, concordance index (C-index), calibration plot and Hosmer–Lemeshow test were used to measure the predictive ability of the nomogram. In order to evaluate the clinical usefulness of the nomogram, decision curve analysis (DCA) was used to calculate the net benefits at different threshold probabilities. SPSS 26.0 was used in all analyses, and graphs were generated using R software. In these analyses, *P* < 0.05 was considered statically significant different.

### Ethics approval and consent to participate

The studies involving human participants were reviewed and approved by Ethics Committee of the First Affiliated Hospital of Chongqing Medical University. All methods were carried out in accordance with relevant guidelines and regulations. Written informed consent for participation was not required for this study in accordance with the national legislation and the institutional requirements.

## Results

### Patient characteristics

A total of 353 patients were included in the analysis (142 males and 211 females). Among them, a total of 163 patients (46.2%) had postoperative PHT, and the mean age at surgery was about 44 years. PHT occurred more frequently in men (52.1% vs. 30.0%, *P* < 0.001), and in patients with advanced ages (43.0 [43.0–55.5] vs. 45.0 [35.0–54.8], *P* = 0.001). In addition, compared with those patients who had normal BP (complete clinical success) after surgery, patients with PHT generally had higher systolic blood pressure (184 [175–200] vs. 180 [166–190], *P* < 0.001), hyper BMI values (BMI ≥ 25, 49.1% vs. 26.8%, *P* < 0.001), longer duration of hypertension (8.0 [3.0–10.0] vs. 3.0 [0.81–7.0], *P* < 0.001), more frequent presence of diabetes mellitus (27.0% vs. 5.8%, *P* < 0.001), HHD (34.4% vs. 16.8%, *P* < 0.001) and RH (39.9% vs. 23.2%, *P* = 0.001), higher DDD of antihypertensive medications (3.6 [2.6–4.4] vs. 3.0 [2.5–4.0], *P* = 0.003), lower preoperative ARR (270 [110–541] vs. 404 [154–674], *P* = 0.002), lower PAC (293 [192–459] vs. 351 [255–533], *P* = 0.006), higher DRC (1.09 [0.50–2.34] vs. 0.79 [0.50–1.79], *P* = 0.045), and lower eGFR (96.0 [75.6–109.2] vs. 107.2 [91.3–119.5], *P* < 0.001), higher serum potassium nadir (2.70 [2.40–3.00] vs. 2.60 [2.16–2.90], *P* = 0.016), and smaller diameter of nodules (1.4 [1.0–1.7] vs. 1.5 [1.0–2.0], *P* = 0.017) (Table 1).

**Table1:** Preoperative clinical and biochemical characteristics of all patients (training + validation group).

Characteristics	Total	Persistent hypertension(n = 163)	Normal blood pressure(n = 190)	*P*
Age, y	44.0 (38.0–55.0)	43.0 (43.0–55.5)	45.0 (35.0–54.8)	0.001
Sex, M/F	142/211 (38.1%)	85/78 (52.1%)	57/133 (30.0%)	< 0.001
SBP, mmHg	180 (170–200)	184 (175–200)	180 (166–190)	< 0.001
DBP, mmHg	110 (100–120)	110 (100–120)	110 (100–120)	0.011
BMI ≥ 25, yes/no	131/222 (37.1%)	80/83 (49.1%)	51/139 (26.8%)	< 0.001
Duration of HT, y	5.0 (1.0–10.0)	8.0 (3.0–10.0)	3.0 (0.81–7.0)	< 0.001
Family history of HT, yes/no	121/232 (34.3%)	63/100 (38.7%)	58/132 (30.5%)	0.136
Diabetes, yes/no	55/298 (21.8%)	44/119 (27.0%)	11/179 (5.8%)	< 0.001
HHD, yes/no	88/265 (24.9%)	56/107 (34.4%)	32/158 (16.8%)	< 0.001
RH, yes/no	109/244 (30.9%)	65/98 (39.9%)	44/146 (23.2%)	0.001
DDD of antihypertensive medication	3.6 (2.6–4.4)	3.6 (2.6–4.4)	3.0 (2.5–4.0)	0.003
ARR, pg·mL^-1^/uIU·mL^-1^	340 (135–618)	270 (110–541)	404 (154–674)	0.002
PAC, pg/mL	326 (214–503)	293 (192–459)	351 (255–533)	0.006
DRC, uIU/mL	0.96 (0.50–2.10)	1.09 (0.50–2.34)	0.79 (0.50–1.79)	0.045
Lowest serum K^+^, mmol/L	2.68 (2.20–300)	2.70 (2.40–3.00)	2.60 (2.16–2.90)	0.016
eGFR, (mL/min/1.73m^2^)	101.2 (82.9–114.4)	96.0 (75.6–109.2)	107.2 (91.3–119.5)	< 0.001
TC, mmol/L	4.03 ± 0.83	4.08 ± 0.90	3.99 ± 0.76	0.297
TG, mmol/L	1.15 (0.80–1.63)	1.33 (0.98–1.89)	0.98 (0.73–1.40)	< 0.001
Diameter of nodules, cm	1.5 (1.0–2.0)	1.4 (1.0–1.7)	1.5 (1.0–2.0)	0.017
Surgical side, L/R	208/145 (58.9%)	98/65 (60.1%)	110/80 (57.9%)	0.752
Total adrenalectomy, yes/no	204/149 (52.0%)	97/66 (59.5%)	107/83 (56.3%)	0.619

*SBP* systolic blood pressure, *DBP* diastolic blood pressure, *HT* hypertension, *HHD* hypertensive heart disease, *RH* resistant hypertension, *DDD* defined daily dose, *ARR* aldosterone/renin ratio, *PAC* plasma aldosterone concentration, *DRC* direct renin concentration, *Lowest serum K*^+^ lowest serum potassium in history, *eGFR* estimated glomerular filtration, *TC* total cholesterol, *TG* triglyceride.

### Validation group characteristics

There were no significant differences between the training group and the validation group in terms of age, gender, DBP, BMI, duration of hypertension, family history of hypertension, presence of diabetes mellitus, presence of HHD, presence of RH, DDD of antihypertensive medication, ARR, PAC, DRC, lowest serum potassium, eGFR, TC, TG, tumor diameter, and surgical side. (Supplementary Table 1).

### Prediction model

Univariate and multivariate logistic regression analysis were used to determine the predictors of postoperative PHT. Univariate analysis showed that age, gender, SBP, DBP, BMI ≥ 25, duration of HT, presence of diabetes mellitus, HHD, DDD of antihypertensive medication, ARR, PAC, lowest serum potassium, eGFR, and diameter of nodules were all related to PHT after surgery (Table 2). On the basis of univariate logistic regression analysis, we employed collinearity and correlation analysis to eliminate some variables (Supplementary figure). Based on previous studies, we retained BMI, duration, and gender as variables in our analysis, as these three factors can be easily obtained in clinical practice. The dosage of antihypertensive medications prior to surgery may not accurately reflect the patients’ regular DDD during the referral process. We excluded it because of the potential risk of bias, although DDD may possess predictive value. Tumor diameter was excluded because it exhibited partial correlation with BMI, duration, and gender. Other variables were also excluded due to the similar reason. In the multivariate analysis (Table 3), five variables (BMI ≥ 25, male, diabetes, ARR, duration of hypertension) were all independent risk factors of the PHT.

**Table 2:** Univariate logistic regression analysis of predictors of hypertension persistence after operation in training group.

Variable	OR (95% CI)	*P*
Age	1.03 (1.0–1.06)	0.013
Male	2.60 (1.53–4.40)	< 0.001
SBP	1.02 (1.01–1.04)	< 0.001
DBP	1.02 (1.01–1.04)	0.005
BMI ≥ 25	3.16 (1.84–5.42)	< 0.001
Duration of HT	1.13 (1.07–1.19)	< 0.001
Diabetes	6.89 (2.74–17.34)	< 0.001
HHD	2.42 (1.33–4.43)	0.004
RH	2.13 (1.24–3.66)	0.060
DDD of antihypertensive medication	1.33 (1.12–1.73)	0.002
ARR	0.42 (0.23–0.75)	0.004
PAC	0.13 (0.04–0.40)	< 0.001
DRC	1.06 (0.94–1.20)	0.347
Lowest serum K^+^	1.62 (1.05–2.50)	0.030
eGFR	0.98 (0.97–0.99)	< 0.001
TG	1.14 (0.94–1.39)	0.178
Diameter of nodule	0.66 (0.46–0.94)	0.022

Data are expressed as odds ratio (OR) (95% CI).

*SBP* systolic blood pressure, *DBP* diastolic blood pressure, *HT* hypertension, *HHD* hypertensive heart disease, *RH* resistant hypertension, *DDD* defined daily dose, *ARR* aldosterone/renin ratio, *PAC* plasma aldosterone concentration, *DRC* direct renin concentration, *Lowest serum K*^+^ lowest serum potassium in history, *eGFR* estimated glomerular filtration, *TG* triglyceride.

**Table3:** Multivariate logistics regression analysis of predictors of hypertension persistence after operation in training group.

Variable	OR (95% CI)	*P*
BMI ≥ 25	2.07 (1.12–3.81)	0.020
Male	2.33 (1.27–4.25)	0.006
Diabetes	3.97 (1.44–10.92)	0.008
ARR	0.46 (0.23–0.92)	0.027
Duration of hypertension	1.12 (1.06–1.19)	< 0.001

Data are expressed as odds ratio (OR) (95% CI).

*ARR* aldosterone/renin ratio.

**Figure 2:**
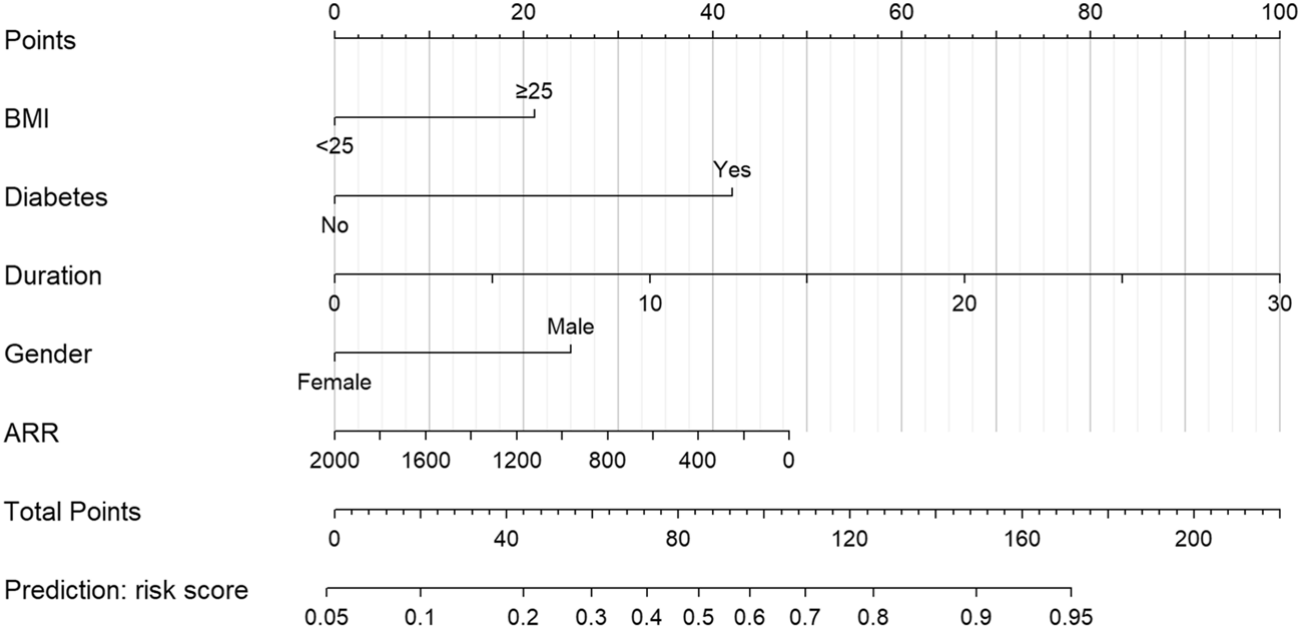
Nomogram for prediction of PHT after operation in PA patients. A line is drawn straight up to the point axis that corresponds with each patient variable to obtain the points. Sum the points of all variables, and draw a line downwards to the risk axis to determine the possibility of PHT.

**Figure 3:**
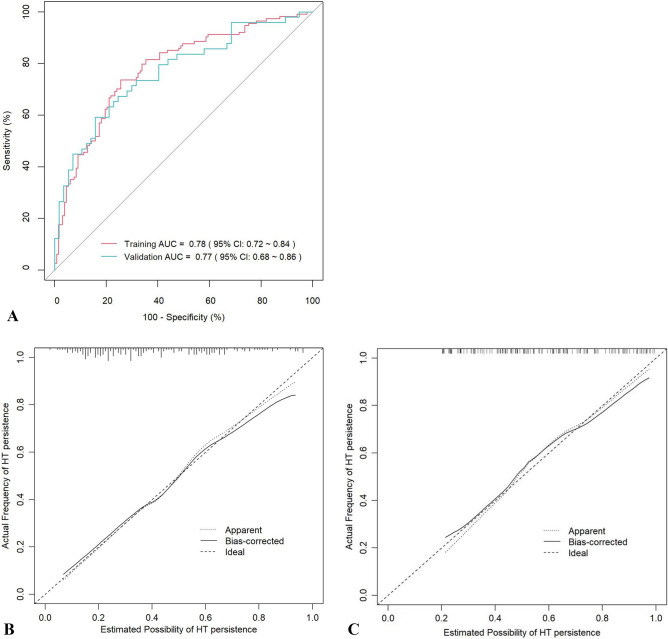
Receiver operating characteristic curves (ROC) and calibration plot of the model. In the training group (**A**), the sensitivity and specificity were 73.7% and 74.4%, with an AUC of 0.78 (95% CI 0.72–0.84). In the validation group (**A**), the sensitivity and specificity were 59.2%% and 84.2%, with an AUC of 0.77 (95% CI 0.68–0.86). The calibration plot depicts the calibration of the model in terms of the agreement between the predicted possibility of PHT and the observed outcomes of PHT. The training group (**B**) and the validation group (**C**) indicated good prediction.

Finally, these five important variables were included to establish a nomogram (Fig. 2). The nomogram showed the score of each variable on each scale. The probability of postoperative PHT was determined by the total score of all variables. According to the model, the ROC curve of the training group and the validation group were drawn. The area under the curve (AUC) of the training group was 0.78 (95% CI 0.72–0.84), and the validation group was 0.77 (95% CI 0.68–0.86) (Fig. 3). The C index of the training group and the validation group were 0.783 and 0.769, respectively.

**Figure 4:**
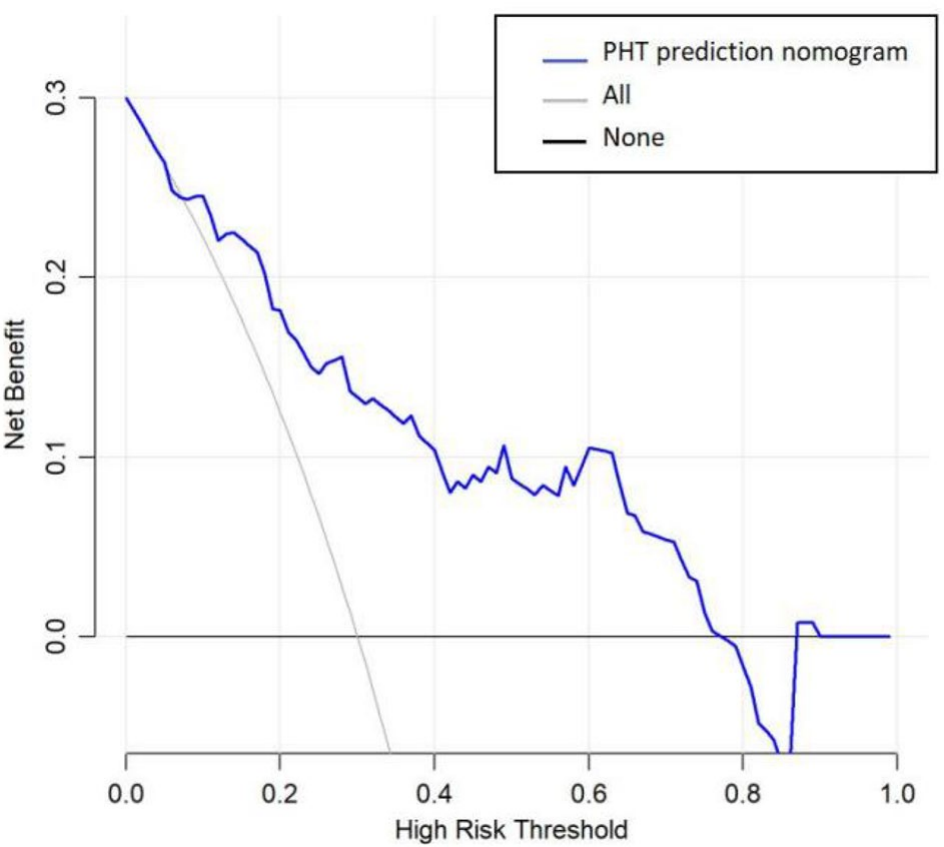
Decision curve analysis (DCA) for the nomogram (in the validation group). The possibility of an individual is denoted as Pi. When Pi reaches a certain threshold (denoted as Pt), it is defined as positive, and intervention (taking antihypertensive medications) may be taken. Therefore, there are benefits (pros) of intervention for patients with post-surgical PHT and harm (cons) of intervention for patients with normal BP after surgery. There is also the harm (cons) of missed intervention for patients with PHT. Pros minus cons is the net benefit. When Pi < Pt, there is no intervention, and the net benefit is 0 (treat-none). When Pi > Pt, all patients receive the intervention, and the net benefit is shown by the gray slanted curve (treat-all). Our DCA indicates that when the threshold probability is approximately 10% to 80%, the use of this predictive model would accrue greater benefit than a treat-all or treat-none strategy.

The calibration plot revealed excellent agreement between the probability of PHT estimated by the nomogram and the actual status of PHT (Fig. 3). The Hosmer–Lemeshow test showed a *P* value of 0.946 in training group and 0.684 in validation group. These results showed that the nomogram is efficient in predicting PHT after surgery. DCA was performed in the validation group, and the results showed that when the threshold probability was about 10% to 80%, the nomogram produced a greater net benefit than a treat-all or treat-none strategy, indicating that the nomogram had good clinical value (Fig. 4).

**Figure 5:**
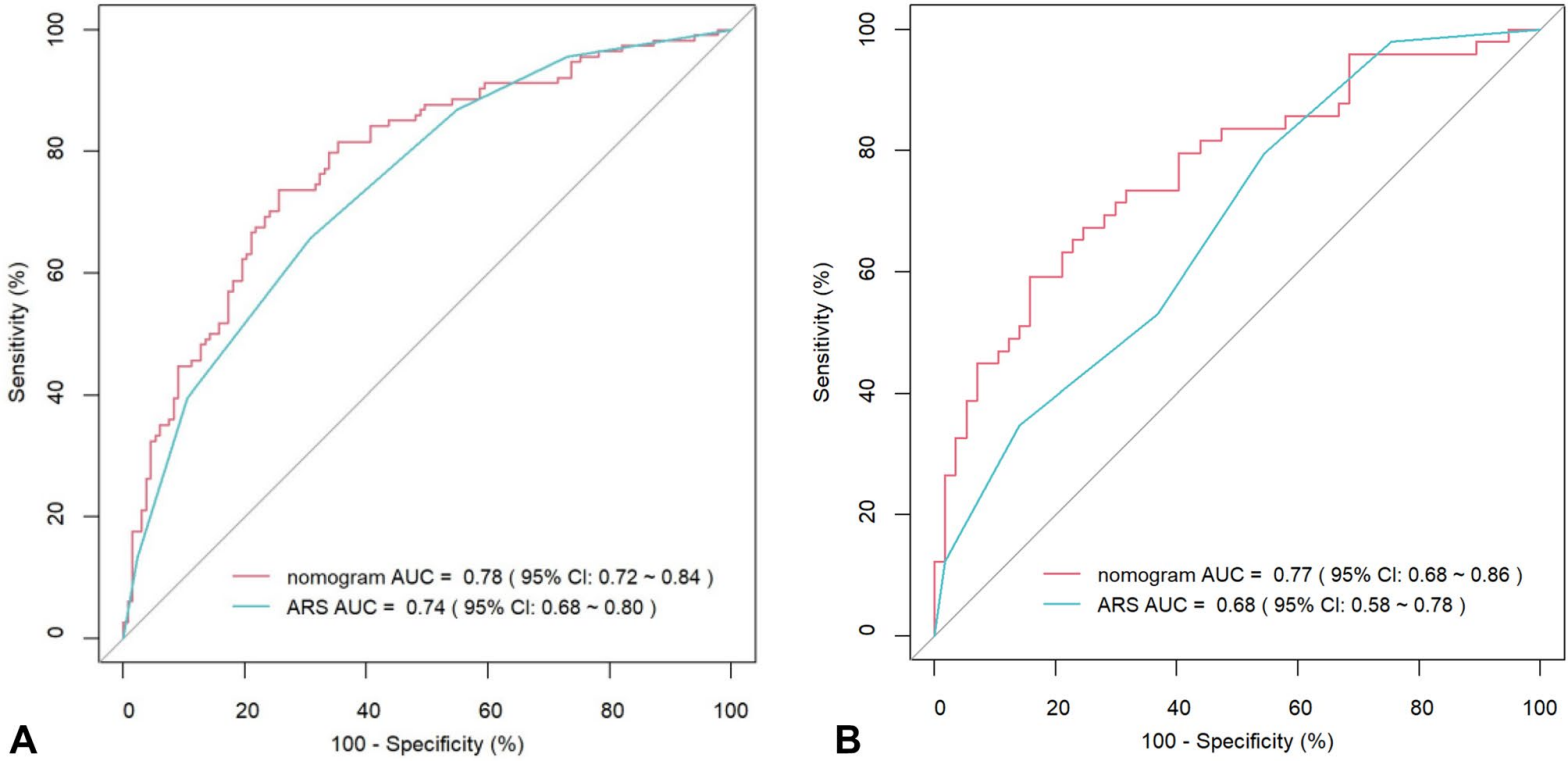
Receiver operating characteristic curves (ROC) comparison of our nomogram prediction model and aldosteronoma resolution score (ARS) in postoperative hypertension outcomes in the training group (**A**) and validation group (**B**).

In addition, we further compared the predictive performance of our model with ARS by ROC. The AUC value of ARS in the training group was 0.74 (95% CI 0.68–0.80), and in the validation group was 0.68 (95% CI 0.58–0.78), both lower than our predictive model (Fig. 5).

## Discussion

Our study found that BMI ≥ 25, male gender, history of diabetes mellitus, ARR, and duration of hypertension were reliable predictors for the PHT postoperatively, and all the variables were easily obtainable. The nomogram based clinical prediction model was useful to predict the PHT postoperatively in patients and identify high-risk patients in order to develop a postoperative follow-up plan. Reported proportions of patients achieving clinical success vary widely between studies (20–66%)^6–10^. The results of our study suggested that 190 patients (53.8%) achieved complete clinical success through surgical treatment, and 163 patients (46.2%) had PHT, which was consistent with previous research reports.

Efforts have been made to establish a predictive model in complete clinical success among PA patients^14–19^. However, the majority of prediction models are focused on predicting clinical success, and those for PHT are lacking. Besides, the prediction indicators for the two outcomes (with or without hypertension) are not entirely identical. For example, previous models have paid less attention to the predictive value of diabetes mellitus absence in relation to postoperative hypertension cure. ARS and PASO score both had a high accuracy in predicting the likelihood of hypertension remission after surgery. ARS score failed to contain several recently reported predictors of surgical outcome, such as ARR^17^. In addition, the AUC of ARS is lower than that of our model, indicating that our predictive model possesses superior predictive ability. In terms of PASO score, although it included target organ damage into their model, not all patients in hypertension centers underwent echocardiography or microalbuminuria measurements, which may limit its clinical applicability^18^. Nevertheless, we have established a high accurate prediction model of PHT after surgery, and all the variables are easily obtained preoperatively.

Long-lasting excessive aldosterone in plasma of PA patients can lead to vascular remodeling, ventricular hypertrophy, and myocardial damage^20–23^. Vascular remodeling may lead to the persistence of hypertension, even if the plasma aldosterone level is normalized by surgery, and the outcome cannot be reversed^24^. We found that the duration of hypertension was an independent risk factor for PHT, and it obtained the highest score in the nomogram. Therefore, early diagnosis and treatment of PA is critical to maximize the benefits of surgery.

Previous studies have shown that higher ARR is related to hypertension remission, but this association was rare shown in multivariate analysis^15^. Our study found that lower preoperative ARR was closely associated with postoperative PHT and was a reliable predictor of postoperative hypertension. Patients with lower preoperative serum potassium and higher ARR are more likely to obtain better clinical outcomes. We speculate that it may be related to the early appearance of clinical symptoms.

PA is closely related to metabolic syndrome^25–27^. Excessive aldosterone or concomitant hypokalemia increases the risk of diabetes. High aldosterone concentration causes impairment of pancreatic islets β cell function and decreases in sensitivity of target organs to insulin^28–30^. In addition, some studies suggested that PA and glucose metabolism may influence with each other^31^. Diabetes complicating PA can increase the progression of cardiovascular events and renal complications^32^. We found that high BMI (≥ 25) and history of diabetes both are independent predictors of the persistence of postoperative hypertension. Previous studies have suggested a potential association between the absence of diabetes and postoperative hypertension resolution^16,33^. Some publications pointed that there is a close association between diabetes mellitus and primary hypertension due to some shared risk factors^32,34–36^. We speculated that patients with the history of diabetes mellitus were more likely to experience postoperative hypertension, possibly due to the vascular changes caused by diabetes. Additional studies should be performed to determine whether diabetes mellitus is a factor that is specific to Asian people or has wider applicability.

Our study aims at identifying patients with PHT, instead of identifying those with postoperative hypertension remission as most models do. This is because these very population (PHT) constitute the majority of the study target (the rate of PHT is 43.2% in this study), but a useful model is lacking. In conclusion, we first established a prediction model for the postoperative PHT. Clinicians could use this nomogram to predict PHT based on clinical and biochemical characteristics, and to strengthen follow-up for high-risk population. Because our present study was a single-center retrospective study, selection deviation is inevitable. In addition, the lack of external validation is a limitation, and relevant prospective multi-center clinical studies should be performed in the future to evaluate the accuracy of the proposed model.

## Supplementary Information

Supplementary Information.

## Data Availability

The datasets used and/or analysed during the current study are available from the corresponding author on reasonable request.

## Abbreviations

PHT: Persistent hypertension
PA: Primary aldosteronism
HT: Hypertension
BP: Blood pressure
SBP: Systolic blood pressure
DBP: Diastolic blood pressure;
HHD: Hypertensive heart disease
RH: Resistant hypertension
ARR: Aldosterone/renin ratio
PAC: Plasma aldosterone concentration
DRC: Direct renin concentration
eGFR: Estimated glomerular filtration
TC: Total cholesterol
TG: Triglyceride
SIT: Saline infusion test
DCA: Decision curve analysis
APA: Aldosterone-producing adenomas
UNAH: Unilateral adrenal hyperplasia
FH: Familial hyperaldosteronism
ACC: Pure aldosterone-producing adrenocortical carcinoma
CT: Computed tomography;
AVS: Adrenal venous samples
OGTT: Oral glucose tolerance test

## References

[1] Xu Z, Yang J, Hu J (2020). Primary aldosteronism in patients in China with recently detected hypertension. J. Am. Coll. Cardiol..

[2] Carey RM, Mantero F, Funder J W (2016). The management of primary aldosteronism: Case detection, diagnosis, and treatment: An endocrine society clinical practice guideline. J. Clin. Endocrinol. Metab..

[3] Williams TA, Lenders JWM, Mulatero P (2017). Outcomes after adrenalectomy for unilateral primary aldosteronism: An international consensus on outcome measures and analysis of remission rates in an international cohort. Lancet Diabetes Endocrinol..

[4] Steichen O, Zinzindohoué F, Plouin PF (2012). Outcomes of adrenalectomy in patients with unilateral primary aldosteronism: A review. Horm. Metab. Res..

[5] Muth A, Ragnarsson O, Johannsson G (2015). Systematic review of surgery and outcomes in patients with primary aldosteronism. Br. J. Surg..

[6] Katabami T, Fukuda H, Tsukiyama H (2019). Clinical and biochemical outcomes after adrenalectomy and medical treatment in patients with unilateral primary aldosteronism. J. Hypertens..

[7] Hannon M, Sze W, Carpenter R (2016). Clinical outcomes following unilateral adrenalectomy in patients with primary aldosteronism. QJM.

[8] Rossi GP, Rossitto G, Amar L (2019). Clinical outcomes of 1625 patients with primary aldosteronism subtyped with adrenal vein sampling. Hypertension.

[9] Picado O, Whitfield BW, Khan ZF (2021). Long-term outcome success after operative treatment for primary aldosteronism. Surgery.

[10] Sellgren F, Koman A, Nordenström E (2020). Outcomes after surgery for unilateral dominant primary aldosteronism in Sweden. World J. Surg..

[11] AMERICAN DIABETES ASSOCIATION (2018). 2 Classification and diagnosis of diabetes: *Standards of medical care in diabetes—2018*. Diabetes Care.

[12] Elliott WJ (2009). Resistant hypertension: diagnosis, evaluation, and treatment: A scientific statement from the American heart association professional education committee of the council for high blood pressure research. Yearb. Cardiol..

[13] Myers GL (2006). Recommendations for improving serum creatinine measurement: A report from the laboratory working group of the national kidney disease education program. Clin. Chem..

[14] Wachtel H, Cerullo I, Bartlett EK (2014). Long-term blood pressure control in patients undergoing adrenalectomy for primary hyperaldosteronism. Surgery.

[15] Yang Y, Williams TA, Song Y (2020). Nomogram-based preoperative score for predicting clinical outcome in unilateral primary aldosteronism. J. Clin. Endocrinol. Metab..

[16] Morisaki M, Kurihara I, Itoh H (2019). Predictors of clinical success after surgery for primary aldosteronism in the Japanese nationwide cohort. J. Endocr. Soc..

[17] Zarnegar R, Young WF, Lee J (2008). The aldosteronoma resolution score. Ann. Surg..

[18] Burrello J, Burrello A, Stowasser M (2020). The primary aldosteronism surgical outcome score for the prediction of clinical outcomes after adrenalectomy for unilateral primary aldosteronism. Ann. Surg..

[19] Development of a Novel Nomogram to Predict Hypertension Cure After Laparoscopic Adrenalectomy in Patients With Primary Aldosteronism [Z/OL]. [2023–06–11]. https://zero.sci-hub.ru/2631/cf1f45ab89373632301a084c87a4a4b9/utsumi2014.pdf#navpanes=0&view=FitH. 10.1007/s00268-014-2612-1 24831672

[20] Diaz-Otero JM, Fisher C, Downs K (2017). Endothelial mineralocorticoid receptor mediates parenchymal arteriole and posterior cerebral artery remodeling during angiotensin II–induced hypertension. Hypertension.

[21] McCurley A, Pires PW, Bender SB (2012). Direct regulation of blood pressure by smooth muscle cell mineralocorticoid receptors. Nat. Med..

[22] Rossi GP, Bolognesi M, Rizzoni D (2008). Vascular remodeling and duration of hypertension predict outcome of adrenalectomy in primary aldosteronism patients. Hypertension.

[23] Tesch GH, Young MJ (2017). Mineralocorticoid receptor signaling as a therapeutic target for renal and cardiac fibrosis. Front. Pharmacol..

[24] Intengan HD, Deng LY, Li JS (1999). Mechanics and composition of human subcutaneous resistance arteries in essential hypertension. Hypertension.

[25] Fallo F, Pilon C, Urbanet R (2012). Primary aldosteronism and metabolic syndrome. Horm. Metab. Res..

[26] Ingelsson E, Pencina MJ, Tofler GH (2007). Multimarker approach to evaluate the incidence of the metabolic syndrome and longitudinal changes in metabolic risk factors. Circulation.

[27] Hannemann A, Meisinger C, Bidlingmaier M (2011). Association of plasma aldosterone with the metabolic syndrome in two German populations. Eur. J. Endocrinol..

[28] Wu VC, Chueh SCJ, Chen L (2017). Risk of new-onset diabetes mellitus in primary aldosteronism. J. Hypertens..

[29] Akehi Y, Yanase T, Motonaga R (2019). High prevalence of diabetes in patients with primary aldosteronism (PA) associated with subclinical hypercortisolism and prediabetes more prevalent in bilateral than unilateral PA: A large, multicenter cohort study in Japan. Diabetes Care.

[30] Adler GK, Murray GR, Turcu AF (2020). Primary aldosteronism decreases insulin secretion and increases insulin clearance in humans. Hypertension.

[31] Hu Y, Zhang J, Liu W (2020). Determining the prevalence of primary aldosteronism in patients with new-onset type 2 diabetes and hypertension. J. Clin. Endocrinol. Metab..

[32] Saiki A, Otsuki M, Tamada D (2020). Diabetes mellitus itself increases cardio-cerebrovascular risk and renal complications in primary aldosteronism. J. Clin. Endocrinol. Metab..

[33] Hartmann I, Hruska F, Hruska F (2022). Hypertension outcomes of adrenalectomy for unilateral primary aldosteronism. Endocrine.

[34] Petrie JR, Guzik TJ, Touyz RM (2018). Diabetes, hypertension, and cardiovascular disease: Clinical insights and vascular mechanisms. Can. J. Cardiol..

[35] Mahler RJ (1990). Diabetes and hypertension. Horm. Metab. Res..

[36] Jia G, Sowers JR (2021). Hypertension in diabetes: An update of basic mechanisms and clinical disease. Hypertension.

