# Supplementary material for: Predictive model for persistent hypertension after surgical intervention of primary aldosteronism
Source: Sci Rep. 2023 Jul 22;13:11868. doi: 10.1038/s41598-023-39028-2 (PMC10363150; doi:10.1038/s41598-023-39028-2)
Supplement: Supplementary file 1 — Supplementary Information. [file 41598_2023_39028_MOESM1_ESM.docx]

**Supplementary methods: Criteria for case detection, confirmation tests and adrenal venous sampling (AVS) interpretation**

The cut-off for ARR detection test is a ARR higher than 2.0 ng·dL−1/mU·L−1, and the cut-off for confirmation tests is a plasma aldosterone concentration (PAC) of 6 ng/dL for the fludrocortisone suppression test (FST), 8 ng/dL for the saline infusion test (SIT) and 11 ng/dL for the captopril challenge test (CCT). For the determination of lateralization, AVS with or without adrenocorticotropic hormone (ACTH) use were both used. For AVS interpretation, the successful cannulation of the adrenal veins was defined as the selectivity index (SI) >2 without ACTH use or SI >3 with ACTH stimulation. The diagnosis of unilateral PA was made if the lateralization index (LI) >2 without ACTH use and LI >4 with ACTH stimulation [1]. In our study, UPA was confirmed by: 1) AVS in 235 patients, or 2) typical adenoma (>1cm) on computed tomography scanning and pathologically diagnosed with adenoma after surgery in 118 patients.

**Supplementary methods: Blood pressure measurement**

In all patients, blood pressure of PA patients were measured in the office or at home, as refer to European Society of Hypertension/European Society of Cardiology guidelines for the management of arterial hypertension [2].

In the office BP measurement, the patient was seated, rested for 3-5 minutes before measurement. BP were recorded in both arms, and the arm that gives the higher reading were used for subsequent readings. Another measurement is required and separate by 2 min. The average of 2 readings were recorded as the blood pressure levels. The patients were also asked if the measurement were similar with the blood pressure at home to exclude white coat hypertension. Blood pressure (BP) were determined using an electronic sphygmomanometer or a mercury sphygmomanometer in all patients in our study. The sphygmomanometers were regularly recalibrated according to manufacturers’ instructions. And the records of office SBP≥140 mmHg and/or DBP≥90mmHg is considered as hypertension.

For home BP evaluation, BP was measured daily on at least 3-4 days and preferably on 7consecutive days. Patient also need a quiet measured room, and was in seated position. After 5 min of rest and with two measurements per occasion taken 1–2 min apart: the results were reported in a standardized logbook immediately after each measurement. Home BP was the average of these readings. And SBP≥140 mmHg and/or DBP≥90mmHg was considered as hypertension in home BP measurement .

**reference**

1. Song Y, Yang S, He W, Hu J, Cheng Q and Wang Y, et al. Confirmatory Tests for the Diagnosis of Primary Aldosteronism. *Hypertension* (2018) 71(1): 118-124. doi:10.1161/HYPERTENSIONAHA.117.10197
2. Mancia G, Fagard R, Narkiewicz K, Redon J, Zanchetti A, Bohm M, et al. 2013 ESH/ESC guidelines for the management of arterial hypertension: the Task Force for the Management of Arterial Hypertension of the European Society of Hypertension (ESH) and of the European Society of Cardiology (ESC). *eart J.* (2013) 34(28): 2159-2219. doi:10.1093/eurheartj/eht151

**Supplement table1. Preoperative clinical and biochemical characteristic(training VS validation group)**

| Characteristics | Total | Training group（n=247） | Validation group（n=106） | P |
| --- | --- | --- | --- | --- |
| Age，y | 47.0（38.0-55.0） | 47.0（39.0-55.0） | 47.0（38.0-55.0） | 0.873 |
| Sex，M/F | 142/211（40.2%） | 94/153（38.1%） | 48/58（45.3%） | 0.250 |
| SBP，mmHg | 180（170-200） | 180（170-200） | 180（160-199） | 0.079 |
| DBP，mmHg | 110（100-120） | 110（100-120） | 110（100-111） | 0.012 |
| BMI≥25，yes/no | 131/222（37.1%） | 89/158（36.0%） | 42/64（39.6%） | 0.603 |
| Duration of HT，y | 5.0（1.0-10.0） | 5.0（1.5-10.0） | 4.0（1.0-10.0） | 0.869 |
| Family history of hypertension，yes/no | 121/232（34.3%） | 84/163（34.0%） | 37/69（34.9%） | 0.968 |
| Diabetes，yes/no | 55/298（15.6%） | 34/213（13.8%） | 21/35（19.8%） | 0.202 |
| HHD，yes/no | 88/265（24.9%） | 59/188（23.9%） | 29/77（27.4%） | 0.587 |
| RH，yes/no | 109/244（30.9%） | 80/167（32.4%） | 29/77（27.4%） | 0.348 |
| DDD of antihypertensive medication | 3.6（2.6-4.4） | 3.6（2.6-4.2） | 3.6（2.3-4.5） | 0.350 |
| ARR，pg·mL-1/uIU·mL-1 | 340（135-618） | 359（138-607） | 287（117-657） | 0.557 |
| PAC，pg/mL | 326（214-503） | 337（234-506） | 300（175-493） | 0.097 |
| PRC，uIU/mL | 0.96（0.50-2.10） | 1.00（0.50-2.09） | 0.78（0.50-2.14） | 0.405 |
| Lowest serum K^＋^，mmol/L | 2.68（2.20-3.00） | 2.60（2.20-2.98） | 2.70（2.27-3.10） | 0.353 |
| eGFR，（mL/min/1.73m^2^） | 101（83-114） | 100（83-115） | 102（84-114） | 0.914 |
| TC，mmol/L | 4.03±0.83 | 4.07±0.83 | 3.94±0.81 | 0.192 |
| TG，mmol/L | 1.15（0.80-1.63） | 1.15（0.79-1.65） | 1.15（0.81-1.60） | 0.972 |
| Diameter of noduler，cm | 1.5（1.0-2.0） | 1.5（1.0-2.0） | 1.5（1.0-2.0） | 0.478 |
| Surgical side, L/R | 208/145（58.9%） | 144/103（58.3%） | 64/42（60.4%） | 0.806 |

**Supplement figure. Collinearity analysis (A) and Correlation analysis (B)**

**
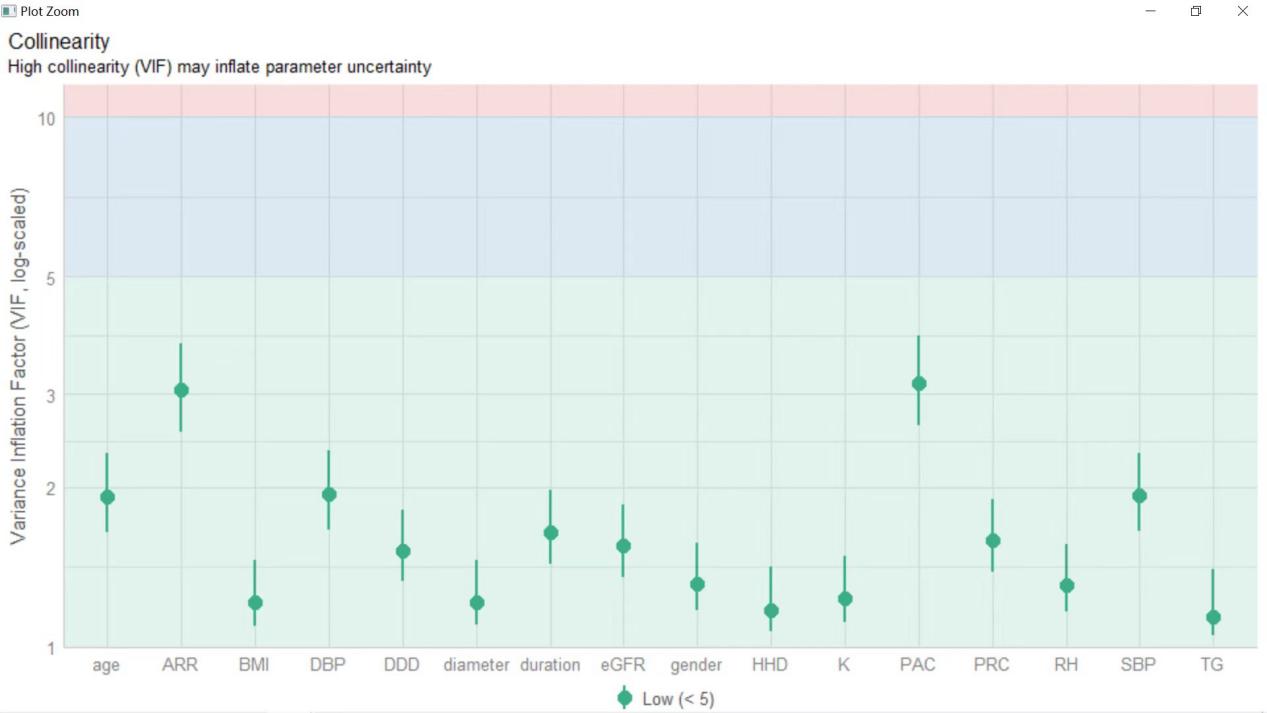
**

**A**


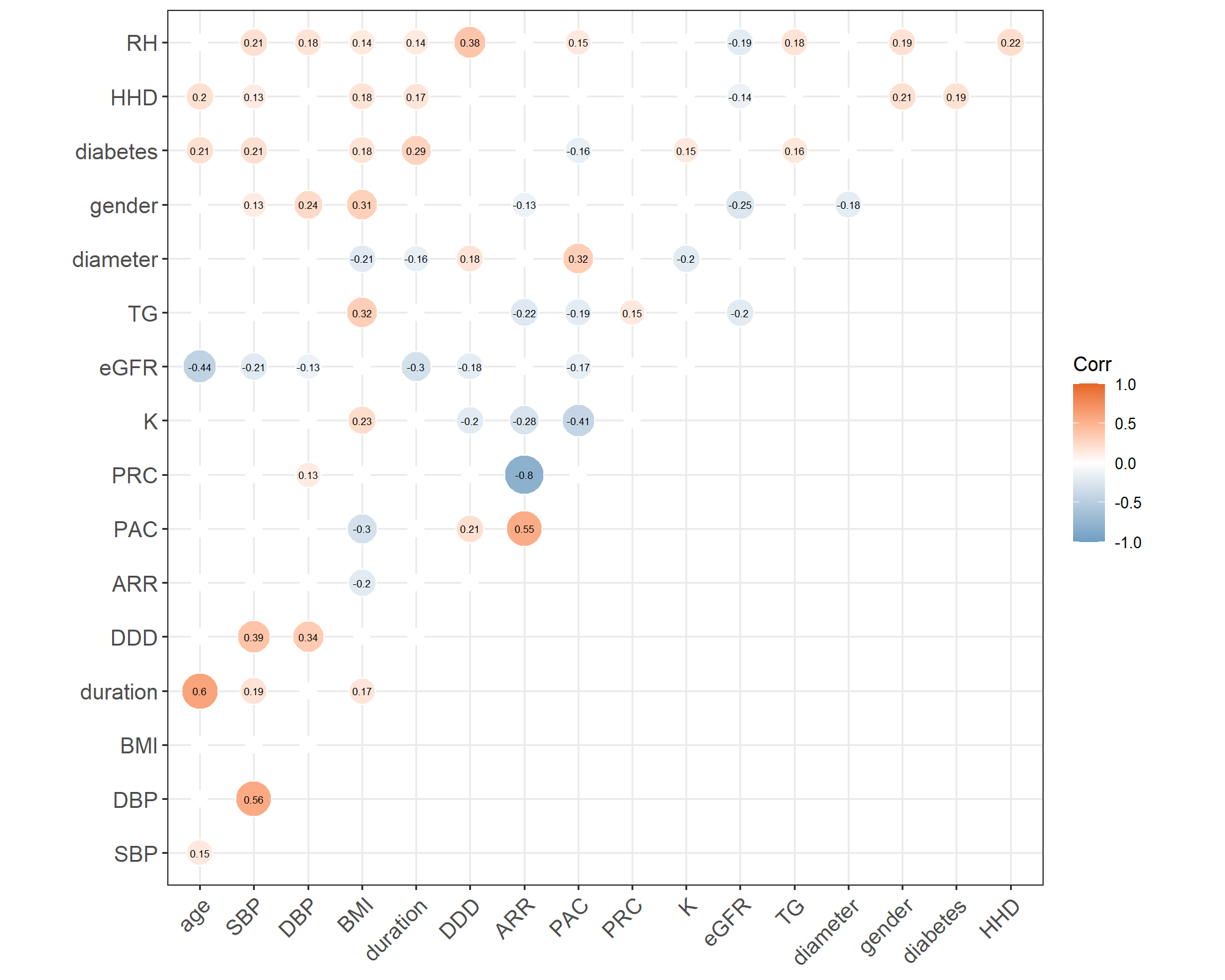


B

Collinearity analysis was conducted to identify potential multicollinearity, where a Variance Inflation Factor (VIF) greater than 2 indicates the possibility of presence of collinearity. Correlation analysis was also performed, and the higher the absolute value of the coordinate intersection between two variables, the stronger the correlation.
